# Tribology of dental materials: from laboratory metrics to clinical durability: review and translational roadmap

**DOI:** 10.3389/froh.2026.1933414

**Published:** 2026-09-22

**Authors:** Naif H. Al-Qahtani, Shahad Alqahtani, Shorooq K. Almajhad, Amjaad Almarzoogi, Noha Taymour

**Affiliations:** 1 Dental Intern, College of Dentistry, Imam Abdulrahman Bin Faisal University, Dammam, Saudi Arabia; 2 Department of Dentistry, King Fahad Medical City, Riyadh, Saudi Arabia; 3 College of Dentistry, Ajman University, Ajman, United Arab Emirates; 4 Doha Medical Complex, Dhahran, Saudi Arabia; 5 Department of Substitutive Dental Sciences, College of Dentistry, Imam Abdulrahman Bin Faisal University, Dammam, Saudi Arabia

**Keywords:** dental tribology, restorative dental materials, wear simulation, clinical wear, tribocorrosion, finite element analysis, machine learning, personalized dentistry

## Abstract

The functional integrity of dental restorations is known by their resilience within the dynamic and hostile tribological ecosystem of the oral cavity. Despite significant advancements in material science, the premature failure of restorations due to wear remains a critical clinical challenge, rooted in a persistent disconnect between controlled laboratory metrics and unpredictable clinical realities. This review provides a comprehensive critical appraisal of dental tribology, delineating the complex interplay of wear mechanisms, attrition, abrasion, erosion, and abfraction, and evaluating the spectrum of current assessment methodologies, from fundamental *in vitro* simulations to advanced clinical quantification. We identified the primary limitations hindering accurate material prediction, specifically the lack of protocol standardization, the inability to replicate synergistic degradation processes such as tribo-corrosion, and the exclusion of patient-specific variables. To bridge this translational gap, we proposed a strategic roadmap integrating multi-tiered testing standardization, finite element analysis, and machine learning to enhance predictive capabilities. Furthermore, we advocated for the development of clinically relevant *in vitro* models that incorporate patient-specific parameters, such as occlusal loading profiles and salivary properties. By shifting the paradigm from descriptive testing to predictive, patient-centric material science, this review outlined a transformative pathway for the development of next-generation restorative materials, ultimately optimizing clinical longevity and treatment outcomes.

## 1 Introduction

In the dynamic ecosystem of the human oral cavity, every bite, every chew, and even the subtle movements of the jaw contribute to a relentless process of wear and tear on natural teeth and their restorative counterparts (1, 2). The longevity and functional integrity of dental restorations, ranging from simple fillings to complex prostheses, are critically dependent on their ability to withstand these constant mechanical and chemical challenges. Indeed, the premature failure of dental restorations due to wear remains a significant clinical and economic burden, impacting patient quality of life and necessitating costly interventions (1–3). This persistent issue underscores a fundamental challenge in modern dentistry: accurately predicting the long-term clinical durability of dental materials based on laboratory assessments (1, 3).

Tribology, the science and engineering of interacting surfaces in relative motion, encompassing friction, lubrication, and wear, is profoundly relevant to the field of dentistry (4–6). The oral environment presents a unique and complex tribological system, characterized by dynamic forces during mastication, the presence of saliva as a lubricant, varying pH levels, temperature fluctuations, and the abrasive nature of food and oral hygiene products (2, 3, 5–7). Understanding the tribological behavior of dental materials and natural tooth structures is paramount for the development of new materials, optimization of restorative techniques, and ultimately, for enhancing patient outcomes (6) Figure 1.

**Figure 1:**
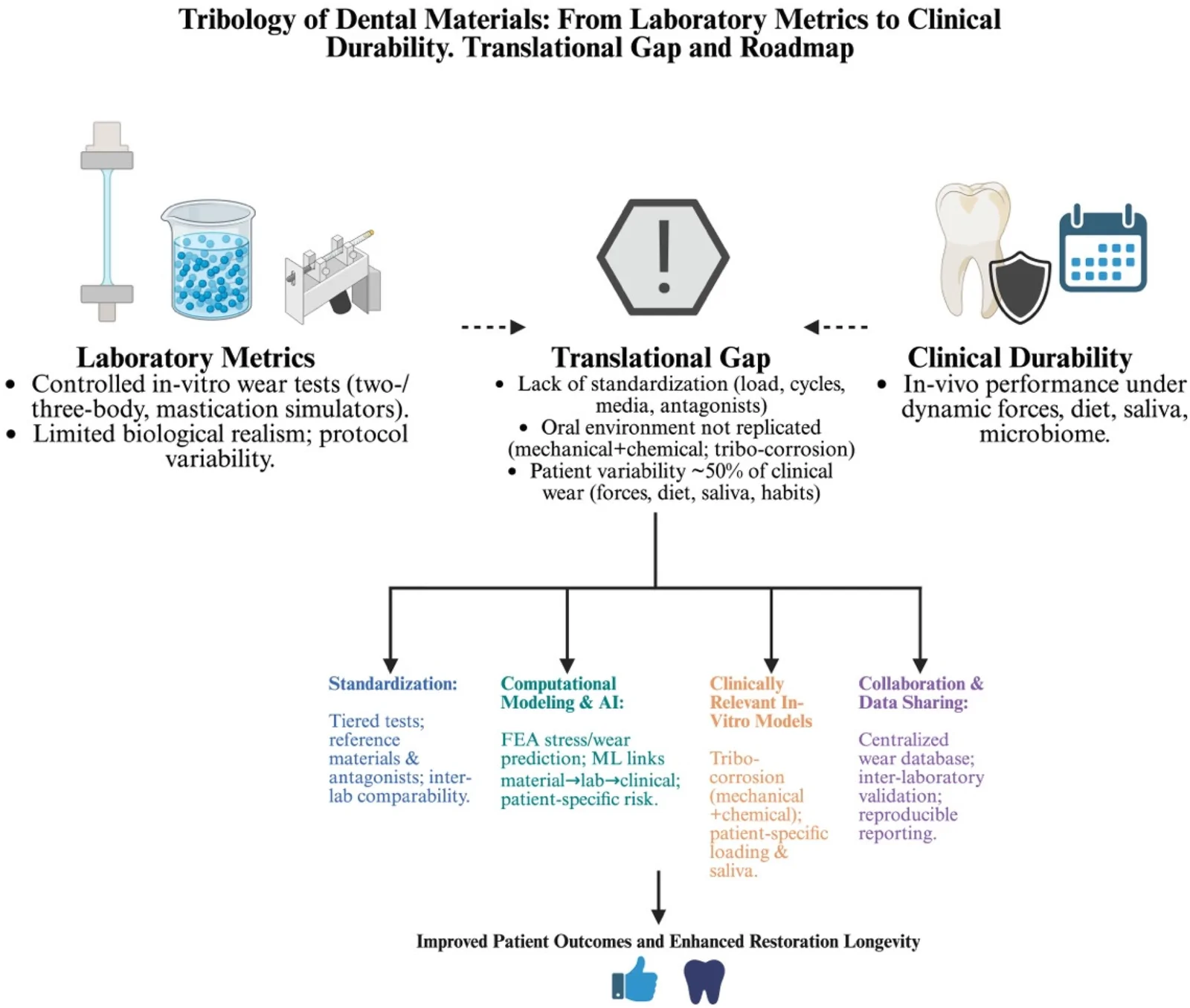
Graphical abstract. Conceptual overview of the translational pathway from laboratory tribometry to clinical durability, highlighting the proposed roadmap integrating standardization, computational modeling, and patient-specific parameters.

Despite decades of intensive research, a persistent disconnect exists between the controlled conditions of laboratory wear testing and the unpredictable realities of the clinical setting (1, 2, 8). While numerous *in vitro* methods have been developed to simulate oral wear, their ability to accurately predict *in vivo* performance remains moderate at best, often failing to account for the multifactorial nature of clinical wear, where patient-specific factors play a dominant role (8). This lack of strong correlation leads to challenges in material selection, hinders the rapid translation of innovative materials from bench to chairside, and contributes to the variability observed in restoration longevity (9).

This review article aims to provide a comprehensive and critical appraisal of the current state of dental tribology, specifically focusing on the journey from laboratory metrics to clinical durability. In doing so, we distinguished between two related but methodologically distinct domains: the tribological degradation of restorative materials, quantified through controlled laboratory tribometry (wear volume, wear depth, friction coefficient), and clinical tooth-surface loss of natural dentition, assessed *in vivo* using validated indices such as the Smith and Knight Tooth Wear Index and the Basic Erosive Wear Examination (BEWE); these domains share overlapping mechanisms but differ in their outcome measures and assessment frameworks, a distinction maintained throughout this review. Unlike prior reviews that address dental wear mechanisms or single testing methods in isolation, this review uniquely integrates laboratory and clinical assessment frameworks with a concrete translational roadmap spanning standardization, computational modeling, and machine learning, and extends this framework to emerging self-healing, bio-inspired, and smart restorative materials. We explored the fundamental mechanisms of wear in the oral environment, critically evaluated the strengths and limitations of existing *in vitro* and clinical assessment methods, and highlighted the significant gaps that impede accurate prediction of material performance. Furthermore, this article proposes a translational roadmap, outlining strategies and future directions to bridge the existing divide, foster greater clinical relevance in research, and ultimately enhance the long-term success of dental restorations. By systematically analyzing the current landscape and charting a course for future endeavors, this review seeks to contribute to a more predictive and patient-centric approach to dental material science.

## 2 Methodology

This article was designed as a narrative review rather than a systematic review or meta-analysis. A broad literature search was conducted using PubMed/MEDLINE, Scopus, Web of Science, and Google Scholar to identify relevant foundational and recent publications addressing dental tribology, restorative material wear, *in vitro* wear simulation, clinical wear assessment, computational modeling, and translational strategies. Search terms included combinations of “dental tribology,” “dental material wear,” “restorative materials,” “*in vitro* wear testing,” “clinical wear,” “tribo-corrosion,” “finite element analysis,” “machine learning,” “artificial intelligence,” and “personalized dentistry.” The literature search was conducted from May 2021 to May 2026, and considered literature published up to June 2026, with older foundational articles included when they provided essential conceptual or methodological background. Two authors (N. Al-Qahtani and N. Taymour) independently screened titles, abstracts, and full-text records against the inclusion and eligibility criteria described below. Disagreements regarding study selection were resolved through discussion with a third author (S. Alqahtani) until consensus was reached. The search initially identified 47 publications; following screening for relevance and eligibility, 3 publications were excluded and 44 met the inclusion criteria, forming the basis of this narrative review. Reference lists of selected articles were also screened to identify additional relevant sources. Because this was not a systematic review, no formal PRISMA protocol, risk-of-bias assessment, or meta-analysis was performed. Articles were included if they addressed dental tribology, restorative material wear, *in vitro* or clinical wear assessment, computational modeling, or translational relevance to restorative material durability. Publications were excluded if they were unrelated to dental materials, lacked clear relevance to wear or tribological behavior, or did not provide sufficient methodological or conceptual contribution to the review topic.

Only publications in the English language were considered; this language restriction was applied for practical reasons of translation and quality assessment and is acknowledged as a potential source of selection bias in Section 8 (Limitations). References were prioritized for inclusion based on (i) direct relevance to dental wear mechanisms, *in vitro* simulation, or clinical assessment; (ii) methodological rigor and journal quality (peer-reviewed, indexed sources); and (iii) recency, with preference given to publications from the last ten years except where older studies provided foundational or still-authoritative concepts (e.g., classic descriptions of attrition, abrasion, and erosion).

To contextualize the growth of this field, a supplementary Scopus search for “dental tribology” and related terms was also performed. Consistent with a prior scoping review of tribology terminology in dental composites research, which identified a marked increase in relevant publications after 2010 (5), Scopus-indexed literature on dental tribology has grown substantially over the past decade, reflecting rising research interest in this field.

## 3 The fundamentals of dental tribology

The oral cavity is a dynamic and hostile environment where teeth and restorative materials are constantly subjected to complex mechanical and chemical challenges (10). Understanding the fundamental principles of tribology, the science of friction, lubrication, and wear, is therefore essential for comprehending the degradation of dental materials and natural tooth structures (1, 10). This section delineates the primary wear mechanisms encountered in the oral environment and the key factors that influence dental wear (5, 10).

### 3.1 Wear mechanisms in the oral environment

Dental wear is a multifactorial process, typically categorized into several distinct mechanisms, often acting synergistically to cause material loss (3). These include:

- Attrition: This refers to the physiological wear of tooth substance or restorative material resulting from direct tooth-to-tooth contact during mastication or parafunctional habits like bruxism (3, 11). Attrition is characterized by smooth, polished facets on occlusal and incisal surfaces (12).
- Abrasion: Abrasion is the mechanical wear of tooth structure or restorative material by foreign substances, such as food particles, abrasive dentifrices, or dental instruments, in contact with the dental surface (3, 13). It can be further classified into two-body abrasion (direct contact between the abrasive and the surface) and three-body abrasion (an abrasive particle trapped between two surfaces in relative motion) (3). Clinically, abrasion often manifests as V-shaped notches at the cervical margins or generalized wear facets (14).
- Erosion: Unlike attrition and abrasion, erosion is the chemical loss of tooth substance or restorative material due not to bacterial action, but to acids of intrinsic (e.g., gastric reflux) or extrinsic (e.g., acidic foods and beverages) origin (3). While primarily chemical, erosion can significantly weaken dental surfaces, making them more susceptible to mechanical wear mechanisms, a phenomenon known as tribo-corrosion or erosive wear (7).
- Abfraction: This is a controversial wear mechanism characterized by wedge-shaped defects at the cervical margins of teeth, often attributed to occlusal forces causing tooth flexure at the cemento-enamel junction (3, 15). The debate continues regarding its distinct etiology vs. being a form of stress-induced abrasion or erosion (15). Because the etiology and diagnostic boundaries of abfraction remain debated, its inclusion in this review should be interpreted as a proposed or controversial mechanism rather than a definitively established wear category equivalent to attrition, abrasion, and erosion.

The synergistic interplay between these mechanisms is crucial. For instance, an eroded surface becomes softer and more prone to abrasive wear, while abrasive wear can expose fresh surfaces to chemical attack. This complex interaction makes the prediction and prevention of dental wear particularly challenging (7) Figure 2.

**Figure 2:**
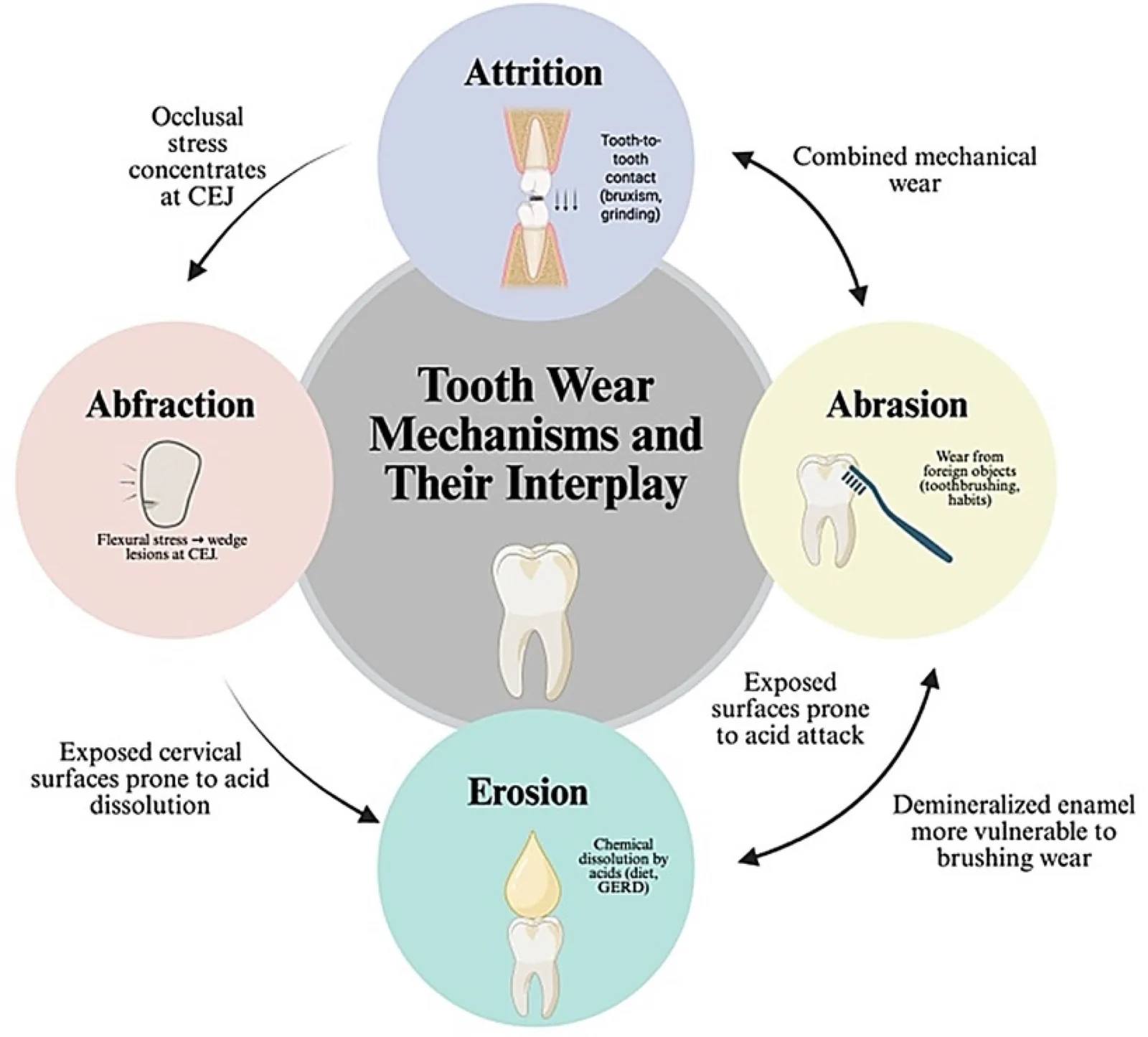
Wear mechanisms in the oral environment: the interrelationship of mechanical and chemical tooth wear processes.

### 3.2 Key factors influencing dental wear

The rate and pattern of dental wear are influenced by a confluence of factors related to the material itself, the patient, and the clinical context:

#### 3.2.1 Material properties:

The intrinsic properties of both natural tooth structure (enamel, dentin) and restorative materials significantly dictate their wear resistance. Key properties include hardness, fracture toughness, elastic modulus, surface roughness, and chemical composition (10). For example, ceramics, while hard, can be brittle and cause significant wear to opposing natural teeth if not properly polished (10). Resin-based composites' wear resistance is influenced by the type, size, shape, and volume fraction of filler particles, as well as the integrity of the resin matrix (3).

#### 3.2.2 Patient-Related factors:

Individual patient characteristics contribute substantially to the variability observed in clinical wear (8). These include:

- Occlusal Forces: The magnitude, frequency, and direction of forces generated during mastication vary widely among individuals. Parafunctional habits like bruxism (teeth grinding or clenching) can generate substantially elevated occlusal forces, with some studies reporting peak values approaching 900 N in severe cases; however, such figures represent upper-range measurements from specific study populations rather than typical or average forces, and considerable inter-individual variability exists, leading to accelerated wear (16).
- Diet: The abrasiveness of food, its rheological properties, and the frequency of consumption of acidic foods and beverages directly impact wear rates (17).
- Salivary Properties: Saliva acts as a natural lubricant, reducing friction and wear. Its flow rate, pH, and buffering capacity also play a role in mitigating erosive challenges and promoting remineralization (18).
- Oral Hygiene Habits: The type of toothbrush, brushing technique, and the abrasiveness of dentifrices can contribute to abrasive wear, particularly on exposed root surfaces or restorative margins (19).

#### 3.2.3 Clinical factors:

The design and execution of dental restorations also influence their tribological performance:

- Restoration Design: The contour, occlusal scheme, and marginal integrity of a restoration can affect stress distribution and contact patterns, thereby influencing wear (20).
- Antagonist Material: The material opposing a restoration is a critical factor. For instance, certain ceramics can be highly abrasive to natural enamel, while others are designed to be more enamel-friendly (20).
- Finishing and Polishing Techniques: A smooth, well-polished surface on a restoration reduces friction and wear, both for the restoration itself and for the opposing tooth structure. Poorly finished surfaces can act as abrasive agents (20).

#### 3.2.4 Biological factors:

Biological determinants also modulate dental wear. Salivary flow rate and composition (already discussed above) are themselves governed by systemic and biological factors such as age, medication use (e.g., xerogenic drugs), and systemic disease (e.g., Sjögren's syndrome), all of which reduce the protective lubricating and buffering capacity of saliva and thereby accelerate wear (2). The oral microbiome and biofilm accumulation can locally alter surface pH and promote demineralization-associated softening of enamel and restorative surfaces, increasing their susceptibility to subsequent mechanical wear (2). Individual differences in periodontal health, tooth position/alignment, and pulp vitality can further modify the local stress and lubrication environment at a given tooth surface (2).

#### 3.2.5 Chemical factors:

Beyond the acid-mediated erosion described above, chemical factors encompass the broader physicochemical environment of the mouth, including salivary pH and buffering capacity, the frequency and duration of exposure to dietary and gastric acids, fluoride and remineralizing agent use, and the chemical stability of the restorative material itself in aqueous, acidic, or enzymatic environments (2). Chemical degradation frequently acts synergistically with mechanical loading, a process termed tribo-corrosion, in which surface softening or leaching of matrix components lowers the effective wear resistance of a material well below what is predicted from purely mechanical wear tests (2).

This complex web of interacting factors underscores the complexity of dental tribology and highlights why laboratory simulations often struggle to fully replicate the clinical reality. The challenge lies in developing testing methodologies that can adequately capture these multifactorial influences to provide more clinically relevant predictions of material durability (20) Figure 3.

**Figure 3:**
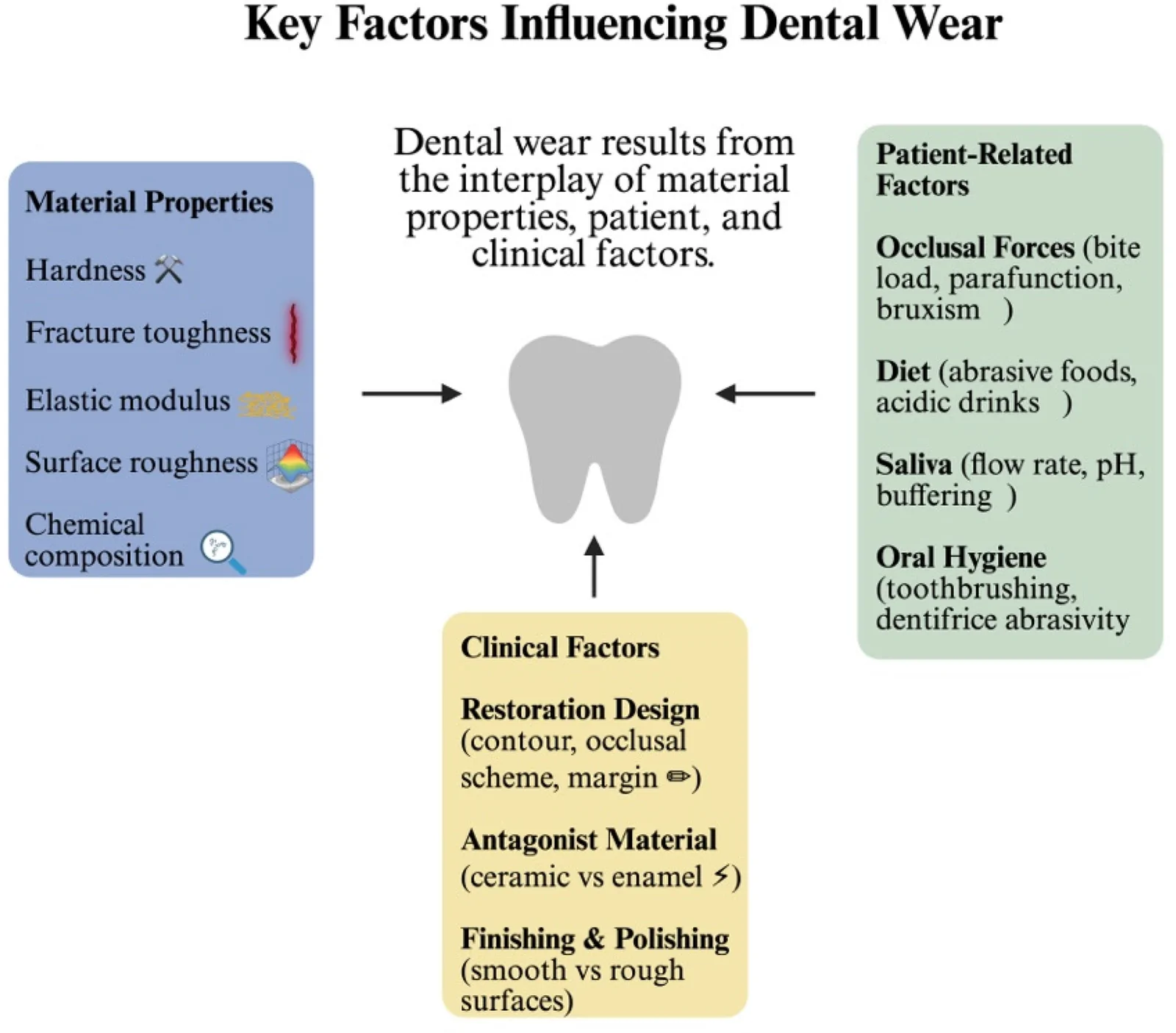
Key factors influencing dental wear.

### 3.3 Classification of dental restorative materials

A material's tribological behavior is fundamentally rooted in its constituent chemistry. For clarity, dental restorative materials can be classified into four broad ingredient-based categories:

- Metals and Alloys: Including noble/high-noble gold alloys, base-metal cobalt-chromium and nickel-chromium alloys, dental amalgam (a mercury-silver-tin-copper alloy), and commercially pure titanium or titanium alloys (e.g., Ti-6Al-4V) used in implants and frameworks; these are characterized by high hardness and ductility, generally producing low wear on themselves but variable wear on opposing enamel depending on surface finish (21).
- Ceramics: Including feldspathic and leucite-reinforced porcelains, lithium disilicate glass-ceramics, and yttria-stabilized tetragonal zirconia polycrystals (Y-TZP); these consist of crystalline and glassy silicate or oxide phases and are hard and wear-resistant but can be highly abrasive to opposing natural enamel if inadequately polished or glazed (22).
- Polymer-Based Composites and Resins: Comprising an organic resin matrix (commonly Bis-GMA, UDMA, TEGDMA) reinforced with inorganic filler particles (silica, glass, zirconia-silica, or ytterbium fluoride) at varying size and loading, bonded via a silane coupling agent; wear resistance is dictated by filler content, particle size/shape, and the integrity of the resin-filler interface (10).
- Cements and Luting Agents**:** Including glass ionomer cements (an acid-base set of fluoroaluminosilicate glass and polyacrylic acid), resin-modified glass ionomers, and resin cements; these are generally softer and more susceptible to erosive and abrasive wear than definitive restorative materials but are clinically important at restoration margins (23). Surface and optical stability of resin cements can also be quantitatively tracked using CIELAB colorimetry and 3D profilometry (24).

## 4 Laboratory assessment of dental material wear: A critical appraisal

The quest for durable and long-lasting dental restorations has led to the development of numerous laboratory methods to assess the wear resistance of dental materials. These *in vitro* tests aim to simulate the complex oral environment and predict the clinical performance of materials before they are used in patients. However, as this section will detail, a significant gap persists between laboratory findings and clinical reality, largely due to the inherent limitations and lack of standardization of current testing protocols (8).

### 4.1 Overview of *in vitro* wear simulation methods

A variety of wear simulation devices are used in dental research, each with its own set of parameters and purported clinical relevance. These can be broadly categorized as follows:

#### 4.1.1 Pin-on-disc/plate testers:

These are among the simplest and most widely used tribometers. A pin (representing a tooth or restorative material) is loaded against a rotating or reciprocating disc or plate (representing the antagonist or an abrasive surface). While useful for fundamental studies of friction and wear, their simplified kinematics and contact geometry limit their clinical relevance (24, 25).

#### 4.1.2 Two-body and three-body wear simulators

These devices attempt to more closely mimic the oral environment (5).

- Two-body wear simulators involve direct contact between the material specimen and an antagonist, simulating attrition (5).
- Three-body wear simulators introduce an abrasive slurry (e.g., food particles, dentifrice) between the specimen and antagonist, simulating abrasion. Several such devices have been developed, including the ACTA, Zurich, Oregon, and Minnesota models, each with unique design features (5).

#### 4.1.3 Mastication simulators

These are the most sophisticated wear testing devices, designed to replicate the complex three-dimensional movements of the mandible during chewing, as well as the forces, temperatures, and chemical environment of the oral cavity. Examples include the “Rub&Roll” device and the “Dento-Munch Robo-Simulator”. While these simulators offer the highest potential for clinical relevance, they are often expensive, complex, and not widely available for cross-center comparisons (7).

#### 4.1.4 Toothbrush abrasion testers:

Reciprocate a bristled brush, typically loaded with a standardized slurry, across a specimen for a defined number of cycles to quantify abrasive wear from oral hygiene procedures; results are commonly reported as relative dentin/material abrasivity (RDA) values (19).

#### 4.1.5 Erosion-abrasion protocols:

Combine cyclic immersion in acidic challenges (e.g., citric acid or cola-type beverages) with mechanical brushing or two-body loading to reproduce the synergistic tribo-corrosive wear seen clinically, and are considered more clinically representative than either challenge alone (7).

#### 4.1.6 Fatigue wear testing:

Subjects specimens to a high number of sub-critical cyclic loads (often >1,000,000 cycles) to assess long-term degradation, microcrack propagation, and eventual bulk or marginal fracture under conditions more representative of years of clinical service than short-duration wear tests (26).

### 4.2 Laboratory characterization and analytical techniques for wear quantification

A rigorous laboratory assessment of wear additionally depends on the analytical techniques used to quantify material loss and characterize the degraded surface, each with distinct working principles and trade-offs Table 1:

**Table 1:** Summary for the characterization techniques for wear quantification.

Technique	Principal application	Advantages	Limitations
Contact Profilometry (27)	Surface roughness, wear depth	Simple, rapid, quantitative	Contact may damage soft surfaces
Non-Contact Optical Profilometry (27)	Wear volume, surface topography	Non-destructive, high resolution, 3D data	Sensitive to surface reflectivity
Confocal Laser Scanning Microscopy (28)	3D surface reconstruction, wear facet morphology	High resolution, optical sectioning	Time-consuming, costlier equipment
Scanning Electron Microscopy (10)	Surface morphology, elemental analysis	High magnification, qualitative/EDS capability	Destructive preparation often required
Atomic Force Microscopy (28)	Nanoscale roughness, nanomechanical properties	Ultra-high resolution, *in situ* capability	Slow scan times, small field of view
Micro-Computed Tomography (29)	Subsurface defects, internal degradation	Non-destructive, 3D internal imaging	Lower resolution than microscopy
Micro-hardness Testing (9)	Indirect wear resistance screening	Rapid, low-cost, widely standardized	Hardness alone does not predict wear

#### 4.2.1 Contact and non-contact profilometry

Stylus (contact) or optical (non-contact, 3D) profilometers scan a surface to quantify vertical wear depth, wear volume, and surface roughness (Ra, Rz) with micrometer-scale resolution; non-contact optical profilometry avoids the risk of stylus-induced surface damage and is now the more widely used approach (27).

#### 4.2.2 Confocal laser scanning microscopy (CLSM)

Provides high-resolution 3D reconstruction of surface topography via optical sectioning, allowing precise quantitative wear volume and depth measurements as well as qualitative assessment of wear-facet morphology, though acquisition and processing are more time-consuming than simple profilometry (28).

#### 4.2.3 Scanning electron microscopy (SEM)

Offers high-magnification qualitative and semi-quantitative imaging of wear-surface morphology (e.g., identifying abrasive grooves, filler protrusion/pull-out in composites, or micro-cracking in ceramics), and is frequently combined with energy-dispersive x-ray spectroscopy (EDS) to assess elemental changes at the worn surface (10).

#### 4.2.4 Atomic force microscopy (AFM)

Enables nanoscale characterization of surface roughness and, in some configurations, nanomechanical properties (e.g., localized hardness/modulus) of a worn surface, offering resolution beyond that achievable with optical methods, at the cost of a much smaller field of view and slower scan times (28).

#### 4.2.5 Micro-computed tomography (micro-CT)

Provides non-destructive, three-dimensional volumetric quantification of material loss and can additionally detect subsurface defects (e.g., microcracks, porosity) that surface-only techniques cannot capture, making it valuable for evaluating both external wear and internal degradation simultaneously (29).

#### 4.2.6 Micro-hardness testing (vickers/knoop):

Indirectly relates to wear resistance by quantifying a material's resistance to localized plastic deformation; while hardness alone does not fully predict wear behavior (fracture toughness and microstructure are equally important), it remains a rapid, low-cost, and widely reported screening parameter across the dental materials literature (9).

### 4.3 Strengths and limitations of current methods

Each wear testing methodology presents a trade-off between simplicity, cost, and clinical relevance. Table 2 summarizes the key characteristics of common *in vitro* wear testing methods:

**Table 2:** Comparison of common *in vitro* wear testing methods.

Method	Parameters	Advantages	Disadvantages	Clinical relevance
Pin-on-Disc/Plate	Load, speed, sliding distance, environment (dry/wet) (10).	Simple, low cost, high reproducibility, good for screening materials and fundamental studies (10).	Simplified contact geometry and kinematics, does not simulate chewing motion, limited ability to incorporate three-body wear (10).	Low. Primarily for ranking materials based on basic wear resistance (10).
Two-Body Wear Simulators	Load, frequency, antagonist material, environment (30).	Simulates direct tooth-to-tooth contact (attrition), more clinically relevant than pin-on-disc (30).	Does not account for abrasive particles (three-body wear), may not replicate complex chewing motions (30).	Moderate. Useful for studying attrition wear of restorative materials against enamel or other materials (30).
Three-Body Wear Simulators	Load, frequency, antagonist material, abrasive slurry (type, concentration), environment (30).	Simulates the effect of food and dentifrice abrasion, can be combined with erosive challenges (30).	Abrasive slurry composition and delivery can be difficult to standardize, may not fully replicate chewing kinematics (30).	Moderate to High. Relevant for assessing the wear of materials in the presence of abrasive media (30).
Mastication Simulators	Complex 3D motion profiles, variable loading cycles, temperature control, artificial saliva (31).	Most closely mimics the clinical situation by simulating chewing motions, forces, and environmental factors (31).	High cost, complex operation, lack of standardization across devices, limited availability for cross-center comparisons (31).	High, though not a formally validated gold standard for *in vitro* wear testing, as results remain difficult to compare between different simulators (31).
Toothbrush Abrasion Testers	Cycle number, load, abrasive slurry composition	Standardized (RDA values), clinically relevant to hygiene-induced wear	Limited to abrasion from hygiene products; does not simulate masticatory wear	Moderate. Relevant for cervical/root surface wear assessment
Erosion-Abrasion Protocols	pH cycling, abrasive challenge, temperature (19).	Simulates synergistic tribo-corrosive wear clinically relevant (19).	Complex protocols, difficult to standardize across laboratories (19).	High (19). Represents clinical reality of dietary acid + mechanical wear (9).
Fatigue Wear Testing	Cyclic loading (>10^6^ cycles), load magnitude (31).	Simulates long-term clinical degradation, microcrack propagation (31).	Time-consuming, does not capture acute wear mechanisms (31).	Moderate to High. Relevant for long-term material durability assessment (31).

### 4.4 Comparative tribological behavior of key restorative materials

To directly address the practical need for cross-material comparison, Table 3 below summarizes the comparative *in vitro* wear performance, principal advantages and limitations, and clinical/therapeutic significance of the restorative material classes most frequently evaluated in the dental tribology literature: resin composites, zirconia, lithium disilicate, hybrid/resin-ceramic CAD-CAM materials, and natural enamel as the clinical reference standard. Broadly, ceramics (zirconia, lithium disilicate) exhibit the lowest self-wear and, when adequately polished, the least abrasive effect on opposing natural enamel, whereas resin composites show greater long-term volumetric wear but are more conservative to place and repair chairside (20, 27, 32, 33). Hybrid CAD-CAM materials occupy an intermediate position, with a modulus of elasticity closer to that of enamel, potentially reducing damage to the antagonist dentition, though long-term clinical wear data for these newer materials remain comparatively limited.

**Table 3:** Comparative wear performance of Key restorative materials.

Material class	Typical wear rate (*in vitro*)	Key advantages	Key limitations	Clinical/therapeutic significance
Resin Composites	Moderate–high; filler-dependent	Esthetic, repairable, conservative preparation	Matrix wear, filler pull-out, lower long-term wear resistance than ceramics (27, 32)	Suitable for low-to-moderate load-bearing restorations; anterior/posterior with regular monitoring
Zirconia (Y-TZP)	Low, especially when polished (not glazed)	High strength/fracture toughness, low wear to opposing enamel when well-polished	Can be highly abrasive if glaze wears off or is under-polished (20)	Preferred for posterior crowns/FPDs and implant-supported prostheses in high-load areas
Lithium Disilicate	Low–moderate	Good esthetics with reasonable strength	Slightly higher antagonist enamel wear than zirconia in some studies (20)	Favoured for anterior/premolar esthetic restorations
Hybrid Ceramics/Resin-Ceramic CAD-CAM Blocks	Moderate	Machinable, more “enamel-friendly” modulus than monolithic ceramics (22)	Long-term wear data more limited than for conventional ceramics or composites	Emerging option for CAD-CAM chairside restorations
Natural Enamel (Reference/Antagonist)	Reference baseline	Physiologic self-lubrication via saliva, biologic remodeling (8)	Irreversible once lost; variable per patient	Serves as the clinical benchmark against which restorative material wear is judged

### 4.5 The gap between *in vitro* data and clinical reality

Despite the increasing sophistication of wear simulation devices, a significant disconnect remains between laboratory data and clinical outcomes. Only a few laboratory wear simulation methods are validated, and they have a moderate correlation with clinical wear and they have estimated that patient-related factors may account for roughly half of the variability observed in clinical wear; this figure should be interpreted as an approximate, order-of-magnitude estimate rather than a precisely validated or universally reproducible statistic, and it likely varies by material class, restoration type, and population studied (8, 34). This discrepancy could be attributed to several factors:

- Lack of Standardization: As highlighted by Maier et al. (2024), there is a severe lack of standardization in testing parameters such as load, frequency, duration, and abrasive media across different studies and devices. This makes it nearly impossible to compare results from different laboratories and contributes to a high degree of variability in wear data, even for the same material (7).
- Inability to Replicate the Full Oral Environment: The oral cavity is a complex ecosystem where multiple degradation mechanisms act synergistically. Most *in vitro* tests fail to capture the intricate interplay between mechanical wear (attrition, abrasion) and chemical degradation (erosion, corrosion), a phenomenon known as tribo-corrosion (7, 34).
- The Dominant Role of Patient-Specific Factors: Perhaps the most significant limitation of *in vitro* testing is its inability to account for the vast variability in patient-related factors. These factors, including occlusal forces, parafunctional habits, diet, salivary composition, and oral hygiene practices, are unique to each individual and are extremely difficult to replicate in a laboratory setting (8).

This gap between the laboratory and the clinic underscores the urgent need for a paradigm shift in how we approach the assessment of dental material durability. The development of more clinically relevant, standardized, and predictive *in vitro* testing methods is a critical step towards this goal (8) Figure 4.

**Figure 4:**
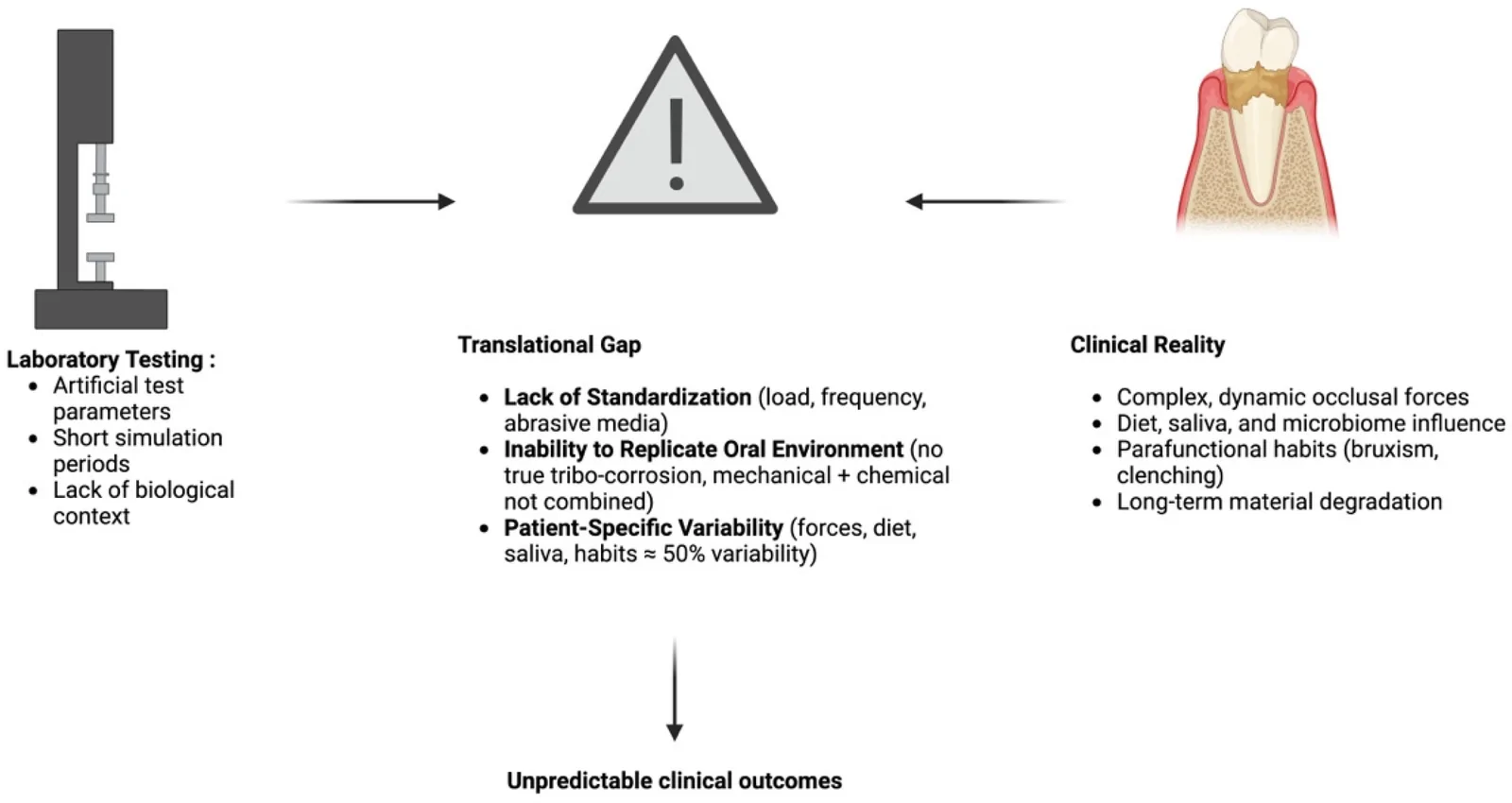
The gap between *in vitro* data and clinical reality: core challenges underlying the disconnect between *in vitro* testing and clinical outcomes.

## 5 Clinical assessment of dental material wear: methods and challenges

Assessing dental material wear in the clinical setting is crucial for understanding the long-term performance of restorations and for validating laboratory findings (35). However, the dynamic and complex nature of the oral environment, coupled with patient-specific variables, presents significant challenges to accurate and standardized clinical wear measurement (35). This section explores the various direct and indirect clinical measurement techniques and discusses the inherent difficulties in obtaining reliable clinical wear data.

### 5.1 Direct and indirect clinical measurement techniques

Clinical wear assessment methods can be broadly categorized into qualitative and quantitative approaches:

- Qualitative Scoring Systems (USPHS/Ryge Criteria): The United States Public Health Service (USPHS) criteria, also known as Ryge criteria, and their subsequent modifications, represent a widely used qualitative method for evaluating the clinical performance of dental restorations, including wear (35). These criteria involve visual and tactile examination of restorations and surrounding tooth structure, assigning scores (Alpha, Bravo, Charlie) based on predefined standards for various parameters such as anatomic contour, marginal adaptation, surface texture, and wear (35). While relatively simple to apply and widely adopted, these methods are subjective, operator-dependent, and often lack the sensitivity to detect subtle or early wear changes (35). They are primarily useful for broad categorization rather than precise quantification of wear (35).
- FDI Criteria: The FDI World Dental Federation criteria (36) represent a more detailed evolution of USPHS/Ryge, scoring restorations across esthetic, functional, and biological domains with greater granularity, and are increasingly preferred in contemporary clinical trials for their improved discriminative ability (36).
- Validated Tooth-Wear Indices: For natural tooth-surface loss specifically, validated indices include the Smith and Knight Tooth Wear Index (37), a five-point scale evaluating wear by surface and depth, and the Basic Erosive Wear Examination (BEWE) (38), a simpler screening index matched to the Basic Periodontal Examination for routine practice use (38).
- Profilometry, Scanner Accuracy, and Minimum Detectable Wear: Contact and non-contact optical profilometry remain the reference standard for measuring surface height loss with sub-micrometer resolution. Intraoral-scanner-based digital subtraction is more clinically practical but less precise: studies using registration-and-subtraction workflows have reported minimum reliably detectable step-height changes of approximately 44 µm on polished enamel and 73 µm on unpolished, freeform natural enamel surfaces (39), with accuracy depending on registration algorithm, reference-area selection, and surface irregularity. Measured changes below these thresholds should be interpreted cautiously, as they may reflect registration error rather than true wear.
- Quantitative Measurement Techniques: To overcome the limitations of qualitative methods, quantitative approaches have been developed, leveraging advancements in imaging and digital technology:
- 3D Scanning and Digital Subtraction Methods: These techniques involve capturing three-dimensional digital models of teeth and restorations at different time points (40). Intraoral scanners or laboratory scanners are used to create highly accurate digital replicas. Subsequent superimposition and digital subtraction of these models allow for precise measurement of material loss (height loss, volume change) over time (40, 41). This approach provides objective and quantitative data on wear progression, offering a more detailed understanding of material degradation *in vivo* Figure 5.

**Figure 5:**
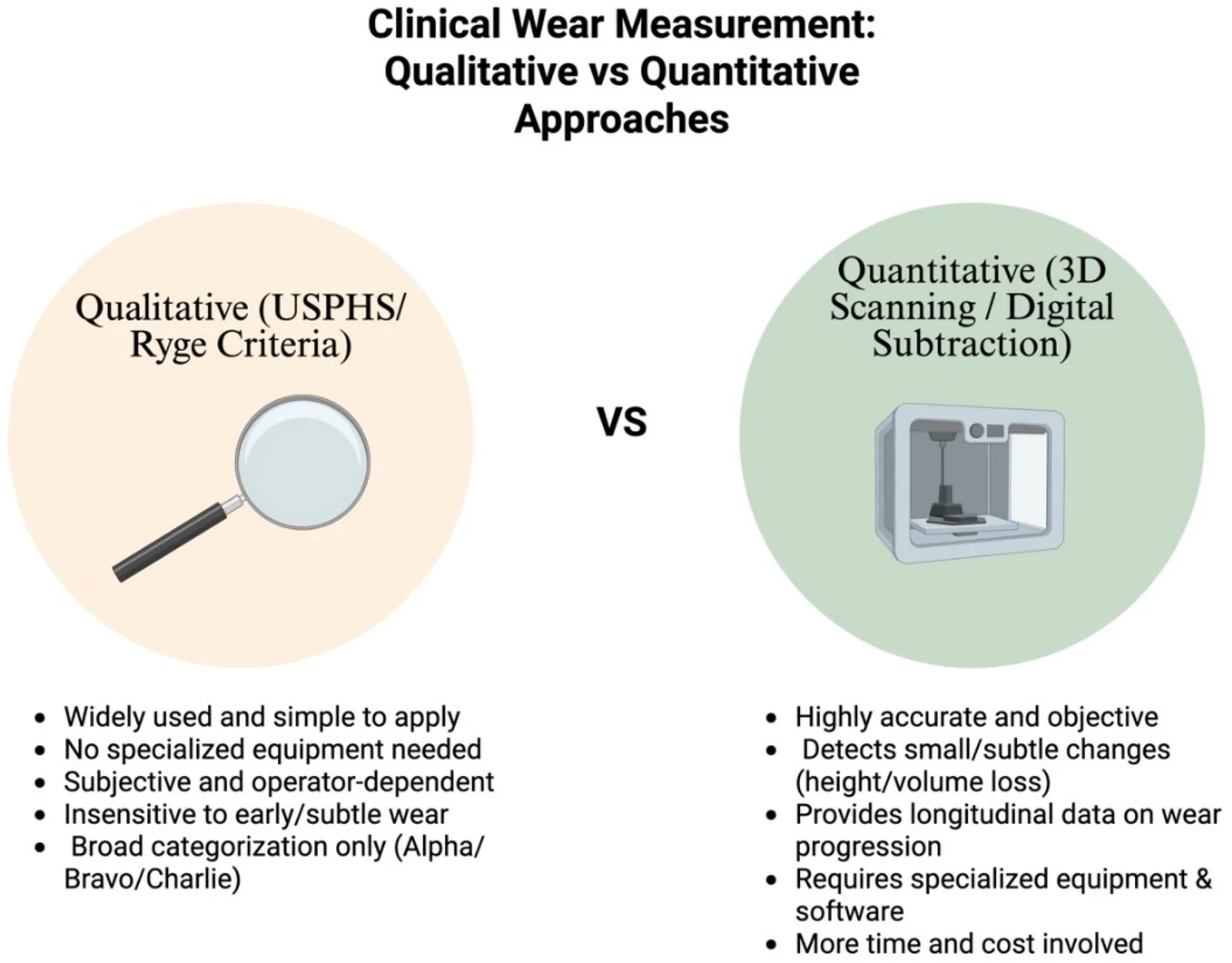
Direct and indirect clinical measurement technique: qualitative (USPHS/ryge) versus quantitative (3D scanning, digital subtraction) approaches to clinical wear assessment.

### 5.2 Challenges in clinical wear measurement

Despite the evolution of clinical assessment methods, several challenges persist that complicate the accurate and consistent measurement of dental wear:

- Lack of Standardization in Clinical Protocols: Similar to *in vitro* testing, there is a notable absence of universally accepted standardized protocols for clinical wear measurement. This leads to variability in study designs, measurement techniques, and reporting, making it difficult to compare results across different clinical trials and studies (8, 42).
- Difficulty in Controlling for Confounding Variables: The oral environment is influenced by a multitude of patient-specific factors that are difficult to control in a clinical study. These include variations in diet, oral hygiene habits, parafunctional activities (e.g., bruxism), salivary flow and composition, and systemic health conditions (8). These confounding variables can significantly impact wear rates and patterns, obscuring the true performance of the dental material itself (32).
- Long-Term Follow-up and Patient Recall Issues: Clinical wear studies often require long observation periods (several years) to capture meaningful wear progression (43). This necessitates consistent patient recall and compliance, which can be challenging to maintain, leading to attrition bias and incomplete data sets (12, 43). The practicalities of conducting long-term clinical research often limit the sample size and generalizability of findings (8, 12, 43).
- Ethical and Practical Considerations: Conducting clinical wear studies involves ethical considerations related to patient burden, radiation exposure (for some imaging techniques), and the need for informed consent (44). Practical limitations, such as chairside time, cost, and the availability of specialized equipment, also influence the feasibility and scope of clinical wear research (44).

The challenges in clinical wear assessment underscore the need for more robust, standardized, and patient-centric methodologies that can provide reliable data to complement and validate laboratory findings, thereby bridging the translational gap in dental tribology (10).

## 6 A translational roadmap: bridging the gap between laboratory and clinic

The persistent disparity between laboratory wear metrics and clinical durability underscores an urgent need for a translational roadmap in dental tribology (7, 27). Bridging this gap requires a multi-faceted approach that encompasses standardization of testing protocols, integration of advanced computational tools, development of more clinically relevant *in vitro* models, and enhanced collaboration within the scientific community (27). This section outlines key strategies to foster a more predictive and clinically applicable understanding of dental material wear Figure 6.

**Figure 6:**
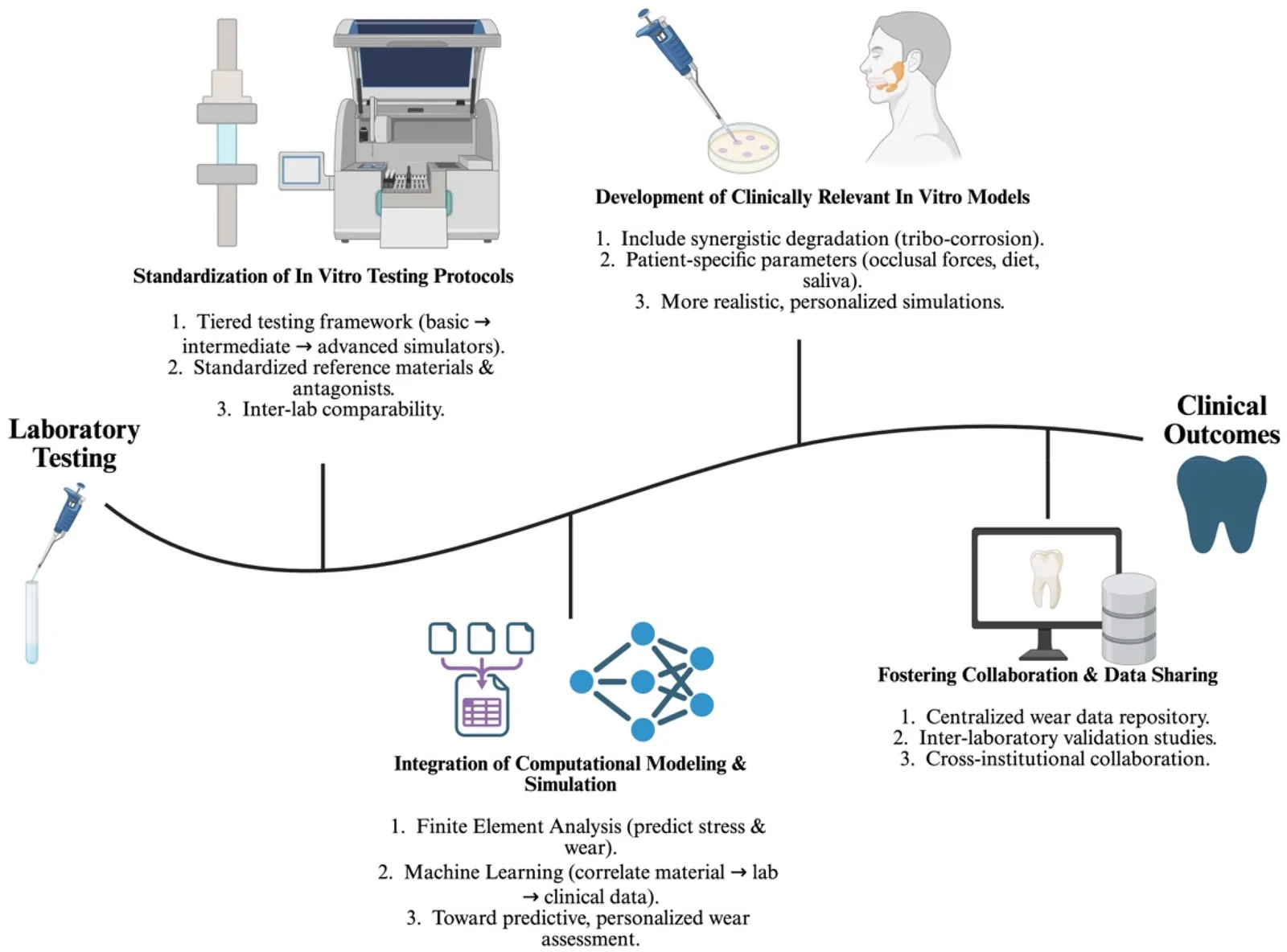
A translational roadmap: bridging the gap between laboratory and clinic.

### 6.1 Standardization of *in vitro* testing protocols

One of the most critical steps towards improving the clinical relevance of laboratory wear data is the establishment of standardized *in vitro* testing protocols. The current variability in testing parameters (e.g., load, frequency, duration, abrasive media, antagonist materials) across different studies makes direct comparison and validation challenging (7, 26, 27). A proposed framework for standardization could involve:

- Multi-level Testing Framework: Implementing a tiered approach to *in vitro* testing, starting with basic screening tests (e.g., modified pin-on-disc) for initial material evaluation, progressing to intermediate simulations (e.g., two- and three-body wear simulators with controlled parameters), and culminating in advanced mastication simulators that closely mimic the oral environment (7, 26, 27). Each level would have clearly defined and universally accepted parameters. For practical implementation, Tier 1 screening tests should clearly report applied load, sliding distance, speed, test medium, specimen geometry, antagonist material, and surface finishing protocol. Tier 2 two-body and three-body wear simulations should additionally standardize antagonist type, loading frequency, number of cycles, abrasive slurry composition, pH, temperature, and storage conditions. Tier 3 mastication simulation should incorporate clinically relevant variables, including multidirectional movement, thermocycling, artificial saliva composition, occlusal loading profiles, and post-test three-dimensional wear quantification.

To operationalize this framework, each tier requires a validation outcome before progression: Tier 1 requires reproducibility (coefficient of variation <15%–20% across ≥3 runs); Tier 2 requires wear-rank concordance with an independent laboratory or reference material; Tier 3 requires benchmarking against a clinically established reference material within pre-specified deviation limits. Progression from Tier 3 to clinical/pilot study should further require testing against a clinically characterized reference material of the same class, with no unexplained divergence in ranking between Tier 2 and Tier 3 results. These thresholds are proposed as a starting point for community standardization, not as validated criteria (7, 26, 27).

- Standardized Reference Materials and Antagonists: The use of certified reference materials and standardized antagonist materials (e.g., human enamel, specific ceramic types) would enable more consistent and comparable results across different laboratories. This would facilitate inter-laboratory validation studies, which are essential for establishing the reproducibility and reliability of wear tests (7, 26, 27).

### 6.2 Integration of computational modeling and simulation

Computational tools offer powerful avenues to complement experimental studies and enhance the predictive capabilities of dental tribology:

- **Finite Element Analysis (FEA**): FEA requires geometrically accurate models (from CBCT/µCT or intraoral scans) and defined material properties (elastic modulus, Poisson's ratio, fracture toughness) as input datasets. Its outputs are stress maps and predicted sites of mechanical concentration mechanistic, not direct wear-volume predictions. Proper validation would compare these outputs against longitudinal *in vivo* wear or fracture data, which remains rare; most published work reports internal consistency (mesh convergence) rather than external clinical validation. Sources of bias include simplified material homogeneity assumptions, idealized rather than true masticatory loading, and small single-center samples. FEA should therefore be regarded as a hypothesis-generating, mechanism-exploration tool, with no demonstrated ability to predict long-term clinical wear (3, 45–47).
- **Machine Learning Models (ML):** ML applications to date rely on small, single-center, heterogeneously reported datasets combining material properties and laboratory wear parameters, less commonly linked to clinical outcomes (45). Typical outputs are wear-rate/friction-coefficient prediction, material ranking, or wear-severity classification from images. Validation is usually internal cross-validation on the same dataset rather than external validation on independent, prospectively collected clinical cohorts, risking overfitting and poor generalizability (46). Bias sources include publication bias toward positive results and inconsistent data-reporting conventions across studies, which hampers pooling of training data (47). ML should currently be described as a potential future decision-support tool, with no demonstrated clinical predictive performance to date (45–47).
- **A critical caveat:** To date, no dental wear-prediction or decision-support AI system has, to our knowledge, been prospectively validated in independent clinical trials with patient-centered outcomes. Existing machine-learning models for predicting wear or mechanical behavior of dental materials (46, 47) have been trained and validated almost exclusively on retrospective or *in vitro* datasets, and none have yet demonstrated prospective clinical validation, real-world deployment, or regulatory clearance as a clinical decision-support tool in dentistry (46, 47). This represents a significant limitation of the current evidence base that should temper expectations regarding near-term clinical translation of AI-based wear prediction.

### 6.3 Development of clinically relevant *in vitro* models

Improving the clinical relevance of *in vitro* models requires a more holistic approach to simulating the oral environment:

- Incorporation of Synergistic Degradation Mechanisms: Future *in vitro* models should move beyond purely mechanical wear to include the synergistic effects of chemical degradation (e.g., pH fluctuations, enzymatic activity) and mechanical loading, a phenomenon known as tribo-corrosion (7, 48–50). This would provide a more accurate representation of the complex degradation processes occurring in the mouth.
- Use of Patient-Specific Parameters: Advanced *in vitro* models could incorporate patient-specific data, such as occlusal loading profiles derived from digital occlusal analysis, dietary habits, and salivary properties (51). This personalized approach would allow for more tailored material testing and a better understanding of individual patient risk factors for wear (51–53).

### 6.4 Fostering collaboration and data sharing

Progress in dental tribology necessitates a collaborative effort across disciplines and institutions:

- Centralized Database for Wear Data: Establishing a publicly accessible, centralized database for both *in vitro* and clinical wear data would enable researchers to pool resources, conduct meta-analyses, and identify trends and correlations that might not be apparent from individual studies. This would also facilitate the development and validation of computational models (7, 9, 31).
- Inter-laboratory Validation Studies: Regular inter-laboratory studies are crucial to ensure the reproducibility and comparability of wear test results. Such initiatives would help refine standardized protocols and build confidence in the predictive power of *in vitro* methods (7, 9, 31).

### 6.5 Practical implications for clinical decision-making and material selection

Translating the roadmap above into practice requires concrete, actionable guidance rather than aspirational goals. For clinicians, the multi-tiered testing and patient-stratification framework proposed here could inform chairside material selection by flagging high-risk patients (e.g., bruxers, those with high dietary acid exposure, or reduced salivary flow) for whom higher-wear-resistance material classes (e.g., monolithic zirconia rather than resin composite) may be prioritized despite higher cost and machine-learning-assisted design optimization (Section 6.2) could shorten the material development cycle and provide clinicians with wear-performance labelling analogous to fatigue or biocompatibility ratings already used for other biomaterials, allowing evidence-based product selection rather than reliance on manufacturer marketing claims alone (54), or conversely, indicate when a more conservative, repairable material is an acceptable trade-off in low-risk patients (54).

### 6.6 Economic impact of improved wear prediction

Improved wear prediction carries substantial economic significance beyond its scientific value (54). Restoration failure due to wear necessitates costly re-treatment, additional chair-time, and, in more severe cases, more invasive and expensive prosthodontic or endodontic intervention following structural failure (54). More accurate, patient-specific prediction of material longevity could reduce the frequency of premature restoration replacement, decreasing direct costs to patients and healthcare systems and indirect costs associated with missed work, treatment-related anxiety, and cumulative loss of tooth structure with each replacement cycle (54). While dedicated health-economic analyses specific to dental-material wear prediction are, to our knowledge, not yet available in the literature, the broader restorative dentistry literature consistently identifies restoration replacement as a dominant driver of lifetime dental treatment cost, underscoring the potential economic value of the predictive, patient-centric paradigm advocated in this review (54); we recommend this as a priority area for dedicated health-economic research (see Section 7).

### 6.7 Emerging and next-generation dental materials: self-healing, bio-inspired, and smart restorative systems

Beyond incremental improvements to conventional material classes, an important translational opportunity lies in next-generation materials engineered to actively resist or respond to wear rather than merely withstand it.

**Self-healing dental resins** incorporating microencapsulated or vascular healing agents, or dynamic covalent/reversible bond chemistries, aim to autonomously repair microcracks before they propagate into clinically significant wear facets or fractures; recently reported high-performance dental resins containing novel starburst-type monomer architectures have demonstrated improved mechanical and potentially wear-relevant performance compared with conventional Bis-GMA/UDMA-based systems, illustrating this direction (55).

**Bio-inspired composites** that mimic the hierarchical, graded microstructure of natural enamel (e.g., aligned mineral-polymer nanostructures) seek to replicate enamel's combination of hardness and fracture resistance more closely than conventional isotropic filler distributions (56). **Smart restorative materials** incorporating stimuli-responsive components (e.g., pH-responsive remineralizing or antibacterial release triggered by cariogenic acid challenge) could, in principle, couple wear resistance with active protection of the tooth-restoration interface, though clinical evidence for their long-term tribological performance remains preliminary (57).

### 6.8 Advanced biocompatible and structurally engineered materials

A complementary translational direction involves biocompatible materials engineered primarily for implant and framework applications but with emerging relevance to wear-critical interfaces.

- **Enhanced and surface-tailored titanium alloys**: Approaches including anodization, plasma spraying, or laser surface texturing aim to improve wear and corrosion resistance at implant-abutment and prosthetic interfaces while preserving osseointegration (21).
- **PEEK (polyetheretherketone) and PEEK-based composites** offer a lower elastic modulus closer to bone and reduced antagonist wear compared with rigid ceramics, though their intrinsic wear resistance under direct occlusal loading is still being characterized (58, 59).
- **Graphene- and graphene-oxide-reinforced dental composites and coatings** have shown promising improvements in mechanical reinforcement, antibacterial activity, and potentially wear resistance in early-stage laboratory studies, while **functionally graded biomaterials,** which vary composition or microstructure continuously across a restoration (e.g., harder occlusal layer transitioning to a more resilient bulk), represent a conceptually attractive but still largely experimental strategy for simultaneously optimizing wear resistance and fracture toughness (56).

**Clinical readiness:** Clinical tribological data for all of these advanced systems remain limited, and their inclusion here is intended to highlight priority areas for future translational research rather than to imply current clinical readiness.

### 6.9 Additively manufactured (3D-printed) dental materials and appliances

Additive manufacturing (3D printing) has emerged as an increasingly important fabrication route for dental appliances and restorative components, introducing a further dimension of mechanical and tribological variability beyond the conventionally processed material classes discussed above. Comparative studies of 3D-printed resins and polymers report tensile modulus, fractal-dimension surface texture, and compression behavior that can differ measurably from conventionally milled or thermoformed materials of nominally the same chemistry (60–62). Thermal aging and simulated clinical use further alter the surface roughness of 3D-printed materials, with implications for both wear susceptibility and biofilm accumulation (61, 63).

Beyond mechanical performance, the biocompatibility and chemical stability of 3D-printed intraoral appliances, including leachable-monomer content and cytotoxicity relative to thermoformed or heat-cured alternatives, remain active areas of investigation, with some studies reporting comparable or superior biocompatibility for 3D-printed materials and others highlighting the need for appliance-specific safety evaluation (61, 64). These findings indicate that additive-manufacturing parameters (resin formulation, print orientation, post-curing protocol) constitute an additional, clinically relevant source of variability that should be considered alongside the material-class comparisons in Section 4.4 when evaluating tribological and biological performance.

## 7 Knowledge gaps and future directions

Looking ahead, the field of dental tribology is poised for transformative advancements that will significantly impact clinical practice. Of the future directions outlined above, we consider the development and multi-center validation of standardized, patient-representative *in vitro* wear-testing protocols (Section 6.1) to be the most important near-term priority. Standardization is a prerequisite for nearly all other goals discussed in this review: without it, computational and machine-learning models (Section 6.2) cannot be trained on comparable data across laboratories, clinically relevant *in vitro* models (Section 6.3) cannot be benchmarked against a common reference, and cross-institutional data sharing (Section 6.4) remains impractical. We therefore recommend that future research efforts, and funding priorities, be directed first toward establishing and validating a consensus, patient-representative wear-testing protocol before further diversifying into computational or materials-development directions.

- Advanced Materials with Enhanced Wear Resistance: Research will continue to focus on developing novel materials with superior tribological properties (1, 3, 10). This includes bio-inspired materials that mimic the hierarchical structure and self-healing capabilities of natural tooth enamel, functionally graded materials with tailored properties, and self-lubricating materials designed to reduce friction and wear in the oral environment (1, 3, 10).
- Personalized Dentistry and Wear Prediction: The integration of advanced diagnostics, such as intraoral scanning and occlusal analysis, with computational modeling and AI may support the future development of patient-specific wear risk assessment tools (40, 65). However, these approaches remain dependent on standardized datasets, clinical validation, and long-term outcome studies before they can be adopted as routine decision-support systems (40, 65).
- The Role of Artificial Intelligence in Dental Tribology: AI and machine learning may offer useful tools for analyzing wear-related datasets, identifying patterns, and supporting future prediction of restoration longevity (45–47). AI-powered image analysis could assist in quantifying wear from clinical scans, while predictive algorithms may help guide material development and restorative design once validated against robust clinical data (45–47). It is important to emphasize that these applications remain in the research domain; clinical deployment requires prospective validation and regulatory approval Figure 7.

**Figure 7:**
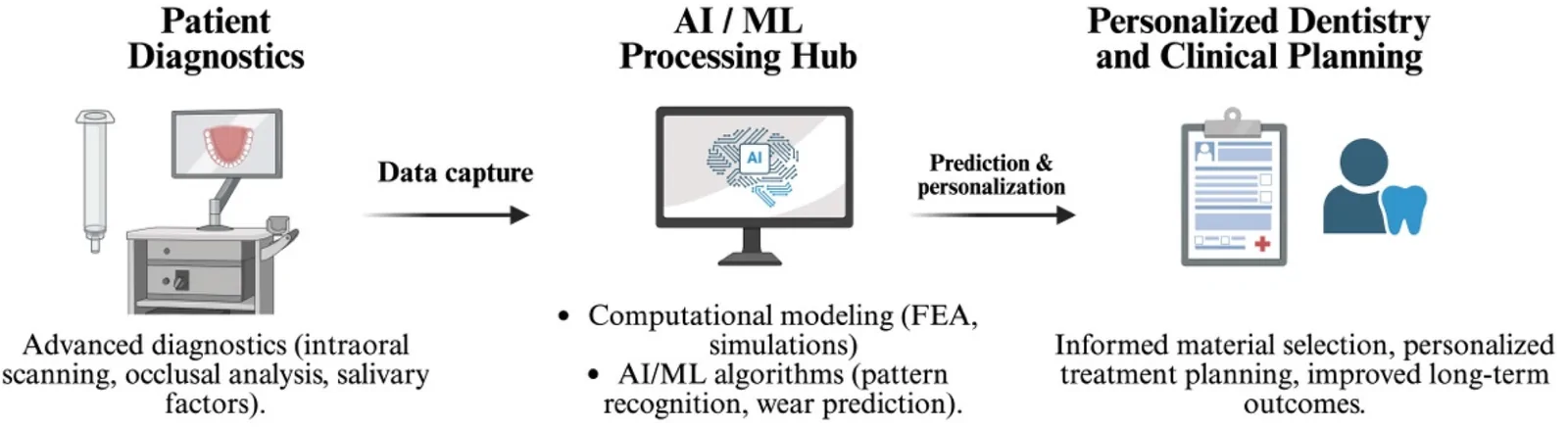
Integration of advanced diagnostics and AI for personalized dentistry and wear prediction.

## 8 Limitations

This review should be interpreted in light of the limitations inherent to narrative reviews. Unlike a systematic review or meta-analysis, this manuscript did not follow a formal PRISMA protocol, perform a structured risk-of-bias assessment, or provide pooled quantitative synthesis. Therefore, selection bias and omission of relevant studies cannot be fully excluded. In addition, the included literature is heterogeneous in terms of material types, wear simulation methods, testing parameters, clinical outcome measures, and follow-up periods, which limits direct comparison across studies. The proposed translational roadmap should therefore be viewed as a conceptual framework rather than an established clinical guideline.

In addition to the limitations already discussed, two further points merit explicit acknowledgment.

1. **AI/Machine Learning Evidence Base:** As noted in Section 6.2, no AI-based dental wear-prediction or decision-support system has yet been prospectively validated in clinical use; all AI/machine-learning evidence discussed in this review is derived from retrospective or *in vitro* data, and the translational roadmap proposed here should be read as a research agenda rather than a description of currently deployed clinical tools.
2. **Language Restriction:** Restriction of the search to English-language publications, together with reliance on a narrative rather than systematic search methodology, may have introduced selection bias and omitted relevant non-English or grey literature; a systematic review or bibliometric mapping study would be a valuable complement to the qualitative synthesis presented here.
3. **Rapidly Evolving Field:** The fields of additive manufacturing, biomaterials, and AI are evolving rapidly; some emerging materials and methods discussed may be superseded by more recent developments during the publication process.
4. **Generalizability:** The findings and recommendations are based largely on literature from developed healthcare systems; generalizability to resource-limited settings may be limited.

## 9 Conclusion

The tribology of dental materials is a complex and multifaceted field, critical to the long-term success and durability of dental restorations. This review has highlighted the intricate interplay of mechanical, chemical, and biological factors that govern wear in the oral environment, emphasizing the significant clinical and economic implications of restoration failure due to wear. While substantial progress has been made in understanding wear mechanisms and developing *in vitro* testing methodologies, a persistent and concerning gap remains between laboratory metrics and the actual clinical durability of dental materials.

Existing *in vitro* wear simulation methods, despite their increasing sophistication, often fall short in fully replicating the dynamic and multifactorial oral environment. The lack of standardization in testing protocols, the inability to account for synergistic degradation mechanisms like tribo-corrosion, and crucially, the failure to adequately incorporate patient-specific variables, contribute to a moderate at best correlation with clinical outcomes. Similarly, clinical assessment of wear, while providing invaluable *in vivo* data, is hampered by challenges related to standardization, control of confounding factors, and the practicalities of long-term patient follow-up.

To bridge this critical translational gap, we have proposed a comprehensive roadmap that includes:

1. **Multi-level standardization** of *in vitro* testing protocols with defined operational parameters and validation milestones.
2. **Integration of computational modeling** (FEA for mechanism exploration) and **machine learning** as long-term research directions, with explicit acknowledgment of current validation limitations.
3. **Development of clinically relevant *in vitro* models** incorporating synergistic degradation mechanisms and patient-specific parameters.
4. **Fostering collaboration** and centralized data sharing to enable robust meta-analyses and model validation.
5. **Translation to clinical practice** through patient stratification frameworks and evidence-based material selection guidance.
6. **Extension to emerging materials** including self-healing, bio-inspired, smart, and additively manufactured systems.

The future of dental tribology lies in embracing these translational strategies. By moving towards more predictive, standardized, and patient-centric research paradigms, we can accelerate the development of advanced, wear-resistant materials, facilitate personalized treatment planning, and ultimately enhance the longevity and success of dental restorations, significantly improving oral health outcomes for patients worldwide. The journey from laboratory metrics to assured clinical durability is challenging, but with concerted effort and innovative approaches, it is an achievable and necessary goal for the advancement of restorative dentistry
